# Development of an ELISA Method to Differentiate Animals Infected with Wild-Type African Swine Fever Viruses and Attenuated HLJ/18-7GD Vaccine Candidate

**DOI:** 10.3390/v14081731

**Published:** 2022-08-06

**Authors:** Lulu Wang, Dan Fu, Weldu Tesfagaber, Fang Li, Weiye Chen, Yuanmao Zhu, Encheng Sun, Wan Wang, Xijun He, Yu Guo, Zhigao Bu, Dongming Zhao

**Affiliations:** 1 State Key Laboratory of Veterinary Biotechnology, National High Containment Facilities for Animal Diseases Control and Prevention, Harbin Veterinary Research Institute, Chinese Academy of Agricultural Sciences, Harbin 150069, China; 2 State Key Laboratory of Medicinal Chemical Biology, College of Pharmacy, Nankai University, Tianjin 300350, China

**Keywords:** African swine fever virus, indirect ELISA, DIVA, p54, CD2v

## Abstract

African swine fever (ASF) is a highly contagious hemorrhagic disease of pigs, posing a significant threat to the world pig industry. Several researchers are investigating the possibilities for developing a safe and efficient vaccine against ASF. In this regard, significant progress has been made and some gene-deleted ASFVs are reported as potential live attenuated vaccines. A seven-gene-deleted live attenuated vaccine candidate HLJ/18-7GD (among which CD2v is included) has been developed in our laboratory and reported to be safe and protective, and it is expected to be commercialized in the near future. There is an urgent need for developing a diagnostic method that can clearly discriminate between wild-type-ASFV-infected and vaccinated animals (DIVA). In the present study, a dual indirect ELISA based on p54 and CD2v proteins was successfully established to specifically distinguish serum antibodies from pigs infected with wild-type ASFV or possessing vaccine immunization. To evaluate the performance of the assay, a total of 433 serum samples from four groups of pigs experimentally infected with the wild-type HLJ/18 ASFV, immunized with the HLJ/18-7GD vaccine candidate, infected with the new lower virulent variant, and specific-pathogen-free pigs were used. Our results showed that the positive rate of immunized serum was 96.54% (p54) and 2.83% (CD2v), and the positive rate of the infection by wild-type virus was 100% (p54) and 97.8% (CD2v). Similarly, the positive rate to infection by the new low-virulent ASFV variant in China was 100% (p54) and 0% (CD2v), indicating the technique was also able to distinguish antibodies from wild-type and the new low-virulent ASFV variant in China. Moreover, no cross-reaction was observed in immune sera from other swine pathogens, such as CSFV, PEDV, PRRSV, HP-PRRSV, PCV2, and PrV. Overall, the developed dual indirect ELISA exhibited high diagnostic sensitivity, specificity, and repeatability and will provide a new approach to differentiate serum antibodies between wild virulent and CD2v-unexpressed ASFV infection, which will play a great role in serological diagnosis and epidemiological monitoring of ASF in the future.

## 1. Introduction

African swine fever (ASF) is a highly lethal hemorrhagic disease of swine caused by African swine fever virus (ASFV), representing a serious hindrance to the development of the pig industry. ASFV is the sole member of the genus *Asfarvirus* within the family *Asfarviridae* [1], and it is a unique and genetically complex virus. The ASFV genome ranges in length from 170 to 194 kb and encodes 150–167 open reading frames (ORFs) depending on the isolates. Morphologically, ASFV is icosahedral in symmetry and has a complex structural architecture composed of several concentric layers [2,3,4].

The disease was first identified in Kenya in the 1920s and spread rapidly in Africa and Eurasia [5,6]. It was then recognized that wild pigs (warthogs and bush pigs) can maintain the virus without showing any clinical signs and act as a chief source of infection for domestic pigs along with some species of soft ticks [5,7]. Since its first discovery, ASF remained an endemic disease in sub-Saharan Africa. However, over the past decades, the disease has spread through many countries of the Caucasus, the Russian Federation, and Central and Eastern Europe, posing a serious risk of further expansion [8]. ASF was first introduced to China in 2018 [9], and the virus was characterized as a highly virulent genotype II ASFV [10,11]. Since then, the disease continues to persist, which poses a significant threat to the pig industry.

Currently, strict quarantine and culling of infected animals is still the main solution to prevent and control ASF outbreaks worldwide. However, as ASF is highly contagious and spreads rapidly among the pig population, it is hard to eradicate only by culling. Thus, an effective vaccine could be the best way to control and eradicate ASF. Different vaccine strategies for ASF have been evaluated in the past decades [12,13,14,15,16]; among them, some gene-deleted attenuated ASFVs showed great promise. In more recent news, Vietnam has made the official announcement that a commercial vaccine against ASFV has been successfully developed, licensed, and will soon be available on the market (https://link.gov.vn/Li4KHPuO, accessed on 13 June 2022). Similarly, a seven-gene-deleted live attenuated ASF vaccine, named HLJ/18-7GD, was successfully developed and proven to be safe and effective in pigs in our laboratory [17]. The vaccine candidate was constructed using the highly virulent wild-type Chinese strain HLJ/18 as a backbone. HLJ/18-7GD was evaluated under clinical trials in five provinces, including Heilongjiang, Henan, the Xinjiang Uygur Autonomous Region, Hebei, and Hubei province, in China, and it is expected to be commercialized in the near future. In this case, to further promote the successful clinical use of the seven-gene-deleted live attenuated ASF vaccine in the field, a diagnostic assay is urgently needed to distinguish the serum antibodies induced by wild-type ASFV infection and vaccine immunization.

Several diagnostic assays have been developed to detect ASF, with reliable results and having their own strengths and weaknesses. These include polymerase chain reaction (real-time and conventional PCR), enzyme-linked immunosorbent assay (ELISA), immunoperoxidase test, immunoblot, immunohistochemistry, immunofluorescent test, and hemadsorption test [18,19,20,21]. ELISA is one of the most sensitive, reliable, and widely used antibody detection assays for ASFV. However, to date, the available ASFV ELISA kits can only detect antibody levels in a sample but cannot differentiate the serum-positive pigs from wild-type virulent ASFV infection or attenuated vaccine candidate immunization. Along with p72, p30, pp62, and p22, p54 belongs to a group of structural proteins, which are immunogenic and often incorporated into vaccine formulations and serological testing [22,23,24,25]. Anti-p54 antibodies appear as early as eight days after infection and persist for several weeks [22,23,26]. In addition, the ASFV-antibody-positive samples remained positive for p54 reactivity after storage for one month at 37 °C [23]. Therefore, p54 represents an ideal antigen target for the detection of ASFV antibodies [26]. CD2v protein encoded by the EP402R gene is directly involved in the hemadsorption phenomenon induced by the infection of susceptible cells with ASFV, and several reports have pointed out that sera from animals either infected with ASFV or immunized with CD2v-recombinant protein can induce specific antibodies against CD2v protein [27,28,29,30]. Moreover, the seven-gene-deleted ASFV vaccine candidate HLJ/18-7GD lacks EP402R gene encoding CD2v protein [17], so the vaccine candidate cannot induce the antibodies against CD2v protein that make CD2v an ideal target for DIVA.

In this study, based on these two proteins (p54 and CD2v), a dual indirect ELISA method for differentiating the serum antibodies of wild-type ASFV infection and CD2v-deleted vaccine immunization was developed and evaluated. The assay has good sensitivity and specificity and will play a great role in ASF diagnosis and serological investigation in the near future in China.

## 2. Materials and Methods

### 2.1. Serum Samples

Serum samples were obtained from four groups of pigs. Group 1: pigs not exposed to African swine fever virus. Sixty-one serum samples were collected from specific-pathogen-free pigs (SPF) and were confirmed to be negative by an ELISA ASFV antibody detection kit (Luoyang Putai Biological Technology Co., Ltd., Luoyang, China). Group 2: pigs experimentally infected with the wild-type HLJ/18 ASFV isolate [9,11]. Forty-six serum samples were collected from pigs that were experimentally inoculated with the wild-type virulent HLJ/18 ASFV. Group 3: pigs vaccinated with the seven-gene-deleted ASFV vaccine candidate (HLJ/18-7GD) [17]. Three hundred eighteen serum samples were obtained from pigs that have been immunized with the seven-gene-deleted ASFV vaccine candidate (HLJ/18-7GD). ASFV-positive samples (from both the wild-type HLJ/18 and the vaccine candidate HLJ/18-7GD) were collected at different time intervals between the years 2019 and 2021 in our laboratory during experimental studies and clinical trials. Accordingly, the status of these samples was determined by the commercial ELISA ASFV antibody detection kit (Luoyang Putai Biological Technology Co., Ltd., China). Group 4: eight serum samples were obtained from pigs experimentally infected with the new low-virulent ASFV strain in China [31]. Of note, the ASFV target protein of the commercial ELISA ASFV antibody detection kit (Luoyang Putai Biological Technology Co., Ltd., China) is p30.

### 2.2. Antigen Preparation 

#### 2.2.1. Expression of p54 Protein 

Initially, p54/E183L (excluding the predicted transmembrane domain) from ASFV HLJ/18 strain (accession. no. MK333180.1) was amplified using the primers listed in Table 1. To facilitate p54 recombinant construction, *Xho*I and *Eco*RV restriction sites were introduced into the sense and antisense primers, respectively, and the recombinant plasmid pET-30a-p54 was constructed. Accordingly, the recombinant plasmid (pET-30a-p54) was transformed to BL21 (DE3) E. coli cells and expressed as described elsewhere [32,33,34,35,36]. P54 protein was expressed as a His-tagged fusion protein and purified with pre-packed His-Tag column (GE, USA) according to the manufacturer’s instructions. The purified protein was then analyzed by SDS-PAGE and Western blot and stored at −80 °C for use as diagnostic antigen in the following assays.

#### 2.2.2. Expression of CD2v Protein

CD2v/EP402R (Y1-K187) gene from ASFV HLJ/18 strain was inserted into the pAcHBM-8His plasmid via *Bam*HI and *Not*I restriction sites to construct a recombinant plasmid pAcHBM-CD2v [22]. Subsequently, the recombinant plasmid (pAcHBM-CD2v) was transfected into Sf9 insect cells using liposome mediation to construct the recombinant virus for protein expression [37]. Briefly, Sf9 insect cells were seeded on a cell culture plate (6-well plate) at a density of 2 × 10^6^ cells/mL, followed with transfection after 2 h, and was replaced with fresh medium after 4–6 h. After 7 days in the 27 °C incubator, the P0 virus was recovered and the recombinant baculovirus CD2v virus was obtained. Hi5 cells were cultured in ESF921 medium to 2 × 10^6^ cells/mL and inoculated with recombinant baculovirus CD2v virus at a ratio of 1/1000 (*v*/*v*). After 72 h of incubation at 27 °C, the cell culture medium was collected, and the supernatant was harvested after centrifugation at 5000 r/min for 15 min. Accordingly, purification was completed with pre-packed His-Tag column (GE, USA) according to the manufacturer’s instructions. Finally, the purity of CD2v protein was analyzed by SDS-PAGE and Western blot and stored at −80 °C for use as diagnostic antigen in the following assays. Moreover, the specificity of the p54 and CD2v proteins was confirmed by Western blotting using African-swine-fever-positive pig serum.

### 2.3. Development of p54-ELISA and CD2v-ELISA

To establish a dual indirect ELISA for ASFV antibody detection, with an ultimate goal of differentiating whether antibodies are from a natural infection or vaccine immunization, the structural p54 and CD2v proteins were used as coating antigens. It was performed as previously described [32,36,38,39]. Initially, the optimal coating concentration of the two proteins, pig serum sample dilution, and secondary antibody dilution (HRP-conjugated IgG) were determined by checkerboard titration. Once optimal concentrations for each reagent were adjusted, it was applied to analyze large number of serum samples. Briefly, each well of 96-well flat bottom polystyrene plates (Corning Costar 42592; USA) was coated with p54 and CD2v antigens diluted in carbonated buffer (pH 9.6) and incubated at 4 °C overnight or 37 °C 2 h. After washing five times with PBST (PBS containing 0.05% Tween-20), the plates were blocked for 30 min at 37 °C with 1% BSA. Plates were then incubated with primary antibodies, added in a volume of 100 μL/well for 30 min at 37 °C. The serum samples were diluted at 1:40 (dilution buffer: 10% rabbit serum in PBS). After washing five times with PBST, the horseradish peroxidase-conjugated anti-pig IgG antibody (Sigma-Aldrich, Burlington, MA, USA) diluted from 1:200,000 in the dilution buffer was added and incubated at 37 °C for 30 min. Following an extensive washing, 50 μL/well of 3,3′,5,5′-Tetramethylbenzidine (TMB) was added and incubated at room temperature for 15 min. The reaction was stopped by adding 50 μL of 2 M sulfuric acid, and the absorbance was measured at 450 nm and read using a spectrophotometer.

### 2.4. Determination of the Cut-Off Value

Once the optimum coating concentration and serum dilution were fixed, 61 known negative serum samples were used to determine the positive/negative cut-off value. All the samples were confirmed to be ASFV-negative by an ELISA ASFV antibody detection kit (Luoyang Putai Biological Technology Co., Ltd., China). The OD_450_ value of these ASFV-negative serum samples obtained with p54-ELISA and CD2v-ELISA were recorded and cut-off value was determined as the mean absorbance of these 61 negative sera plus three times standard deviation (3 SDs). Serum sample showing OD_450_ value greater than this cut-off value was considered as ASFV-seropositive.

### 2.5. Establishment and Standardization of Indirect ELISA

To assess the accuracy of the developed indirect ELISA, a total of 425 pig serum samples from three groups (sera from experimentally infected pigs with wild-type ASFV virus, sera collected after vaccination with the seven-gene-deleted ASFV vaccine candidate, and negative sera from SPF pigs) were used and tested by p54-ELISA and CD2v-ELISA. Simultaneously, all the samples were also tested by a commercial ELISA kit, which is based on recombinant p30 protein as antigen (Luoyang Putai Biological Technology Co., Ltd., China) for a comparison and as a reference method as well.

The results of these two ELISA methods were compared, and the diagnostic sensitivity and specificity of the test were calculated to evaluate the precision of the indirect ELISA. In this study, we defined sensitivity as the ratio of positive tests from the developed indirect ELISA to the positive tests by the commercial ELISA kit (Luoyang Putai Biological Technology Co., Ltd., China), and specificity was defined as the ratio of negative tests from the developed ELISA to the negative tests (Luoyang Putai Biological Technology Co., Ltd., China).

### 2.6. Serum Cross-Reactivity of Indirect ELISA to Other Pathogens

The cross-reactivity of ELISA was evaluated by testing anti-sera to other swine pathogens, namely porcine reproductive and respiratory syndrome (PRRS), highly pathogenic porcine reproductive and respiratory syndrome (HP-PRRSV), classical swine fever virus (CSFV), pseudorabies virus (PrV), porcine epidemic diarrhea virus (PEDV), and porcine circovirus type 2 (PCV2). All anti-sera were detected and certified by Harbin Guo Sheng Biological Testing Technology Co., Ltd., Harbin, China.

### 2.7. Calculations and Statistical Analysis

ELISA results were exported to Microsoft Excel and basic calculations, including means and SDs, were obtained. The optical densities of the positive control (OD positive) as well as the optical densities of the samples (OD sample) were corrected by subtracting the optical densities of the negative control (OD negative). Percentage positive (PP) of test and reference sera were calculated using the following formula:

$$S/P=\frac{\mathrm{OD}\;\mathrm{sample}\;-\;\mathrm{OD}\;\mathrm{negative}}{\mathrm{OD}\;\mathrm{positive}\;-\;\mathrm{OD}\;\mathrm{negative}}$$

Moreover, interactive dot plot diagram was performed via Graph Pad Prism version 8.0.2. (GraphPad Software Inc., La Jolla, CA, USA).

## 3. Results

### 3.1. The Expression, Purification, and Identification of the Recombinant Proteins

**Figure 1:**
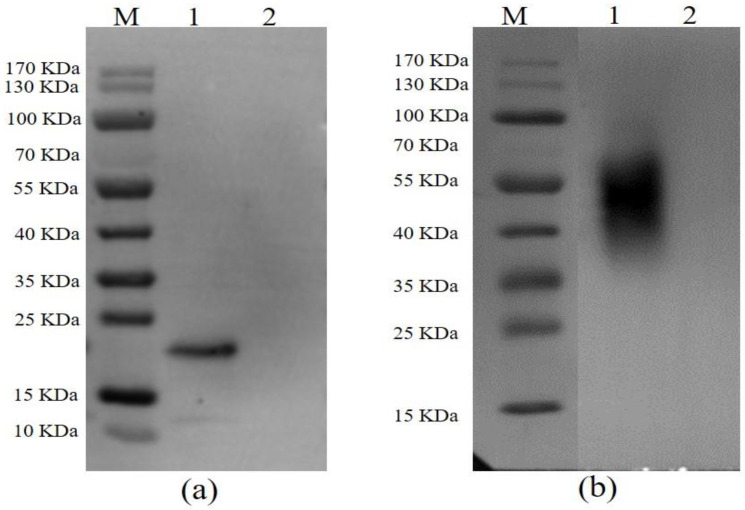
Western blot analysis of p54 (**a**) and CD2v (**b**) with pig anti-ASFV polyclonal antibody. In both figures, lane M is protein molecular weight marker, lane 1 shows the purified fusion protein, and lane 2 is pET-30a empty vector used as a control.

After following the procedures for the expression and purification system as described above, both p54 and CD2v were successfully expressed and purified. The immunoreactivity of proteins to ASFV-seropositive sera was also examined by Western blotting. An expected band revealed that p54 protein was specifically bound by pig anti-ASFV polyclonal antibody (Figure 1a) and CD2v was predicted to be glycosylated as mentioned elsewhere [40,41]. As expected, a glycosylated CD2v protein band was visualized after staining with pig anti-ASFV polyclonal antibody (Figure 1b).

### 3.2. Optimization of Indirect ELISA

Based on the checkerboard titration results, the following reaction conditions were chosen: the optimal coated antigen of p54 protein was measured at 0.07 μg/well, 100 μL/well, and CD2v was adjusted at 0.02 μg/well, 100 μL/well, and optimal serum sample dilution was 1:40. The optimum sample diluent buffer was 10% rabbit serum and 2% Tween-20 in PBS. Finally, the best working dilution of the HRP-goat anti-pig IgG was found to be 1:200,000.

### 3.3. Determination of the Cut-Off Threshold Value

To determine the cut-off values of the indirect ELISA, 61 ASFV-seronegative samples, verified by ELISA ASFV antibody detection kit (Luoyang Putai Biological Technology Co., Ltd., China), were tested by the newly established p54-ELISA and CD2v-ELISA. The average optical density of these negative serum samples (N) was found to be 0.17, and the standard deviation (SD) of these samples was 0.09 for both p54 and CD2v ELISA. Consequently, the cut-off values of the indirect ELISAs were calculated to be 0.40 (mean OD value of negative samples plus three SDs). Likewise, serum samples showing an S/P value ≥ 0.40 were interpreted as ASFV-seropositive and vice versa. A serum sample was judged as positive for wild-type ASFV (wild-type infection) if it was positive to both p54 and CD2v antigens, whereas it was considered as vaccine-immune-positive if p54 antibody was positive and CD2v antibody was negative. On the other hand, it was regarded as negative when the test sample was negative to both p54 and CD2v antibodies.

### 3.4. Validation of Cross-Reactivity and Sensitivity of p54 and CD2v Indirect ELISA

**Figure 2:**
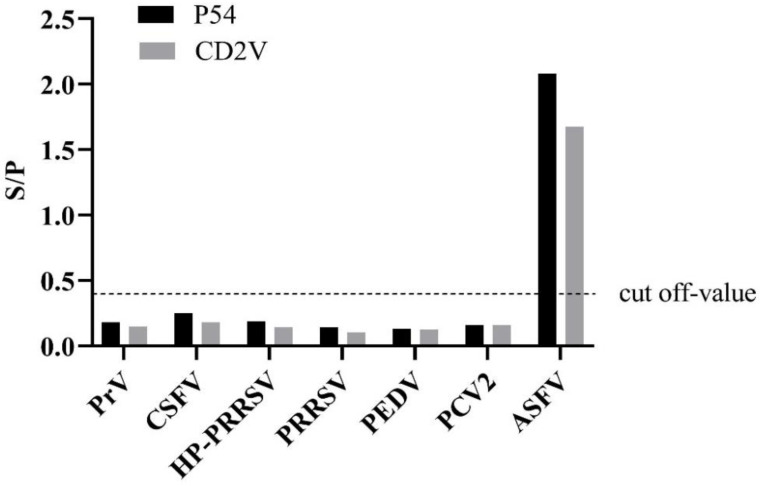
Specificity test of the dual indirect ELISA method. Only ASFV-positive serums show an OD value greater than the cut-off value.

To validate the cross-reactivity, the indirect ELISA was utilized to test other porcine-serum-positive samples (Figure 2). The results showed that these serum samples were ASFV-seronegative and did not cross-react with the indirect ELISA coated with p54 and CD2v, indicating that the established ELISAs were an effective method for detecting and distinguishing ASFV antibodies.

**Figure 3:**
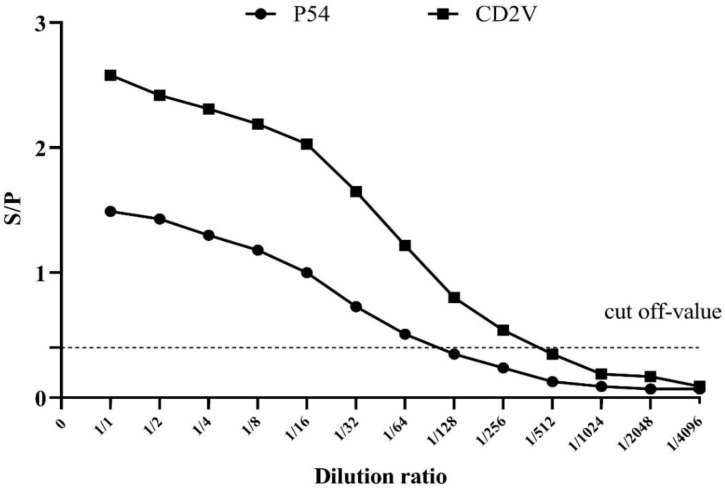
Determining the detection limit of the dual indirect ELISA method. The end point titers of p54-ELISA (solid circles) and CD2v-ELISA (solid squares) are shown. The cut-off value is indicated by the horizontal broken line.

Similarly, in order to determine the detection limit, the sensitivity of the indirect p54-ELISA and CD2v-ELISA were evaluated on serial two-fold dilutions of positive serum samples (Figure 3).

### 3.5. Performance Assessment of the Established Indirect ELISAs

**Figure 4:**
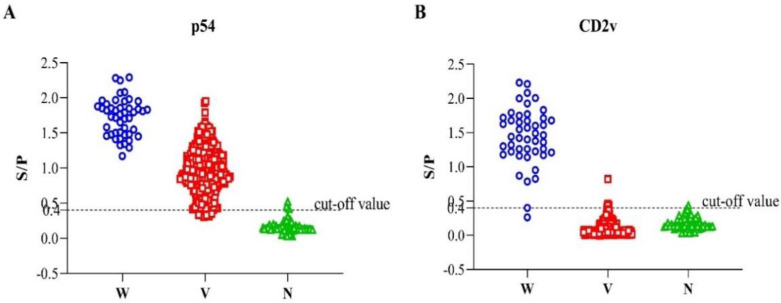
Interactive dot diagram of the dual indirect ELISA; p54-ELISA (**A**) and CD2v-ELISA (**B**) via Graph Pad Prism version 8.0.2. (GraphPad Software Inc., La Jolla, CA, USA). In the graph, the data of positive and negative samples are displayed as dots on three vertical axes (W = sera from experimental infection with wild-type ASFV (HLJ/18); V = immunological sera from pigs vaccinated with the seven-gene-deleted attenuated ASFV vaccine (HLJ/18-7GD) and N = ASFV-seronegative).

To evaluate the performance of the developed indirect ELISA of p54 and CD2v, a total of 425 serum samples were used (Figure 4). The serum samples were grouped as ASFV-seropositive and ASFV-seronegative based on the commercial ASFV antibody detection ELISA kit, of which 364 samples were ASFV-seropositive and 61 were ASFV-seronegative. Out of the total 425 samples, our newly established p54-ELISA detected 357 ASFV-seropositive and 68 ASFV-seronegative. The sensitivity of the p54 indirect ELISA was 96.54% (95% confidence interval: 93.29% to 97.76%) among the seropositive individuals, whereas the specificity of the test was 93.44% (95% confidence interval: 84.05% to 98.18%) among the seronegative groups compared to the commercial ASFV antibody detection ELISA kit, used as a standard evaluation method in this study.

The seven-gene-deleted live attenuated ASF vaccine candidate HLJ/18-7GD lacks EP402R-gene-encoding CD2v protein, so serum samples collected after immunizing pigs with this vaccine should be seronegative to CD2v-indirect ELISA. For this purpose, the ASFV-seropositive serum samples mentioned above were further grouped into samples obtained from vaccinated pigs (*n* = 318) and samples from infection with the wild-type virulent ASFV virus (*n* = 46) based on their known source. The samples obtained from pigs vaccinated with the seven-gene-deleted live attenuated ASF vaccine candidate (*n* = 318) plus the samples that were seronegative by the standard method (*n* = 61) were hypothesized to be CD2v-seronegative. As expected, 369 (369/379) serum samples were found to be CD2v-seronegative by indirect CD2v-ELISA. On the other hand, when the serum samples from infection with the wild-type ASFV virus (*n* = 46) were tested by the indirect CD2v- ELISA, it detects 45 samples as ASFV-seropositive. Hence, the sensitivity of the CD2v-ELISA was 97.83% (95% confidence interval: 88.47% to 99.94%), and the diagnostic specificity was 97.36% (95% confidence interval: 95.31% to 98.85%).

### 3.6. Inter- and Intra-Assay Variation of p54 and CD2v Indirect ELISA

Intra- and inter-assay variability of the p54 and CD2v indirect ELISA were assessed by testing 10 samples, of which five were known ASFV-seropositive and five were known ASFV-seronegative. To calculate the inter-assay variation (reproducibility), the OD values of these ten swine sera were measured five times on ELISA plates coated at different times (comparison among ELISA plates). The analysis produced a coefficient of variation ranging from 1.9 to 13.6%, with an average value of 8.6% (Table 2). For intra-assay variation (repeatability), the OD values of five replicates of the same serum samples were measured on the same plate (comparison within one ELISA plate) and CVs values ranging from 1.8 to 10.3% with an average value of 5.3% were yielded (Table 2).

### 3.7. Assessing the Ability of the Established ELISA to Differentiate the Infection of the Naturally Occurring Lower Virulent ASFV Isolate in China

**Figure 5:**
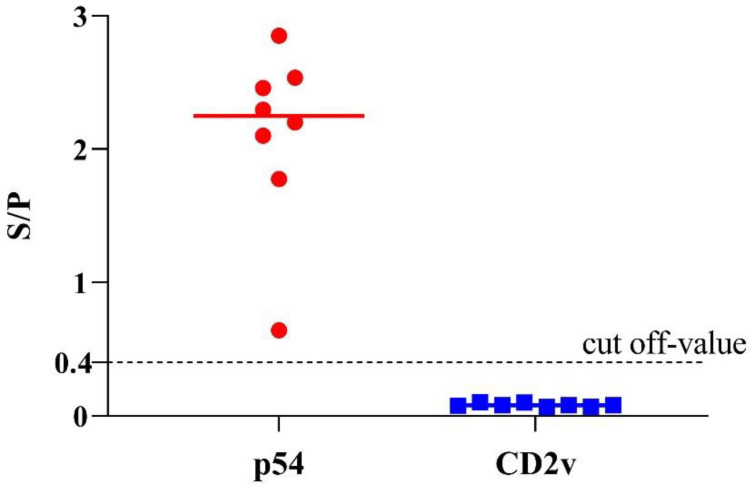
Detection of eight serum samples obtained from pigs infected with the new low-virulent ASFV strain in China. The data of eight positive samples are shown as dots on two vertical axes (1 = p54-ELISA and 2 = CD2v-ELISA).

In a recent study, we have confirmed the emergence of lower virulent natural variants of ASFV strains in China [31], which brings a new challenge to the ongoing control of ASF. These strains had some natural mutations or short-segment deletion in the EP402R/CD2v gene, preventing the viruses from translating intact CD2v protein. Thus, these low-virulent isolates could not elicit antibodies against the ASFV CD2v protein. Here, we investigated the detection ability of p54-ELSA and CD2v-ELISA to these low-virulent ASFV strains in China. Briefly, eight positive serum samples from the infection of these new low-virulent ASFV strain HLJ/HRB1/20 samples were tested by the dual indirect ELISA. As expected, all the samples were positive to p54-ELISA and negative to CD2v-ELISA (Figure 5), indicating this method could also be valuable to differentiate serum antibodies from wild-type virulent ASFV and ΔCD2v (CD2v-unexpressed lower-virulent variants in China).

## 4. Discussion

Currently, the global situation of ASF among pig-producing countries is alarming, and highly sensitive and specific diagnostic tests are critical [42]. Rapid and effective diagnosis is important for limiting the spread of a disease and implementing appropriate measures as quickly as possible [43]. Similarly, vaccinations are highly effective methods of preventing a wide range of animal diseases [44], which is why the focus on ASFV vaccine development is rising due to the global attention of the disease. Recently, some gene-deleted ASFVs have been reported as potential live attenuated vaccines [13,15,16,17,45,46,47]. An interesting piece of news has emerged from Vietnam regarding the commercialization of I177L-deleted ASFV vaccine for field use (https://link.gov.vn/Li4KHPuO, accessed on 13 June 2022), which is a new breakthrough event in the ASFV development era. Equally, the seven-gene-deleted live attenuated ASF vaccine (HLJ/18-7GD) [17] is one of the most promising vaccine candidates as it is claimed to be safe and effective and might be commercialized in the near future. Therefore, ASFV-seropositive pigs either from natural infection with wild-type virulent virus or vaccine immunization must be differentiated in the future. In this regard, paired serum sample testing could be beneficial to discriminate between vaccination and natural exposure.

There are several appropriate diagnostic tests for ASF that provide confident detection of the disease in any epidemiological situation [42,43]. Among them, different kinds of ELISAs and lateral flow assays are easy to use, highly specific, inexpensive, and they possess the ability to detect ASF during the early stages of infection [42,48,49]. However, ASF diagnosis is still complex due to the wide range of clinical forms, naturally occurring CD2v-unexpressed lower virulent strains [31], and the similarity of its symptoms to those of other viral infections, such as classical swine fever (CSF) [20]. Currently, ASF test kits that can differentiate among the wild-type and ΔCD2v (CD2v-unexpressed lower virulent or CD2v-deleted vaccine candidates) ASFV infection are unavailable. Moreover, antibody detection in surviving carrier animals or those immunized with a gene-deleted vaccine is essential for proving the freedom of the disease within the specified areas. Therefore, monitoring ASFV-specific antibodies is of great significance for determining the infection status of the host and evaluating the vaccine effect in the future, especially in countries where ASFV vaccines will be used routinely.

In the present study, we described the production and expression system of two recombinant ASFV proteins (p54/E183L and CD2v/EP402), and these two proteins were used for the development of a dual indirect ELISA with an ultimate goal of differentiating serum antibodies induced by wild-type virulent ASFV infection and CD2v-deleted HLJ/18-7GD vaccination. As stated by Pérez-Filgueira et al., indirect ELISA using p30 antigen expressed by baculovirus demonstrates higher sensitivity than that of conventional ELISA, which uses whole live virus [50], indicating p30 is one of the most antigenic ASFV proteins that is suited to develop a serological diagnosis. Considering this fact, we used a commercial ASF antibody detection ELISA kit (Luoyang Putai Biological Technology Co., Ltd., China) that was developed by targeting p30 ASFV protein as a standard evaluating method for our techniques.

While developing a diagnostic assay, it is often necessary to evaluate the sensitivity and specificity using relative standards of comparison, either from experimentally infected or vaccinated animals [51]. Here, we estimated the sensitivity, specificity, and accuracy of the newly established dual ELISA by testing a total of 433 serum samples from swine with a known background. Out of the total samples, p54-ELSA was able to detect ASFV antibodies in serum samples collected from wild-type infection (46/46), vaccine immunization (311/318), and CD2v-unexpressed lower virulent infection (8/8). Meanwhile, CD2v-ELISA only reacted with ASFV seropositive samples from wild-type infection (45/46). Thus, the significance of our results is that we developed a dual ELISA that can effectively discriminate the antibodies induced by wild-type virulent ASFV infection from the seven-gene-deleted live attenuated ASF vaccine candidate HLJ/18-7GD immunization. Additionally, the intra- and inter-assay variability tests proved that this ELISA method had excellent repeatability (acceptable CV value is <20%) (Table 2) [51].

Therefore, the newly established indirect ELISA method can be explored as a commercial test to distinguish the antibodies induced by wild-type virulent ASFV infection and the seven-gene-deleted live attenuated ASF vaccine candidate HLJ/18-7GD immunization. However, the emergence of naturally occurring lower virulent African swine fever viruses provides a major challenge for these methods since some mutations or deletions occurred in EP402R genes encoding CD2v proteins of these viruses, resulting in the early abortion of intact CD2v protein translation [31]. Thus, to validate whether the developed methods could be used to differentiate wild ASFV infection and naturally occurring lower virulent African swine fever viruses, eight sera from pigs infected with the low-virulent ASFV variant HLJ/HRB1/20 in China were examined by the newly established dual ELISA. Interestingly, all the samples were ASFV-seropositive to p54-ELISA and ASFV-seronegative to CD2v-ELISA (Figure 5), suggesting the diagnostic method is also appropriate for differentiating a wild-type and ΔCD2v infection. Similar to our findings, a dual ELISA based on p30 and CD2v has been developed and published recently [52], indicating other proteins, such as p30 and p72, could also be used together with CD2v for developing a differential diagnosis among wild and ΔCD2v infection.

In conclusion, the HLJ/18-7GD ASFV vaccine candidate has been proven to be safe and protective. However, its commercialization may still take time as it needs to pass various regulatory authorities. Therefore, currently, our method is suitable for distinguishing wild virulent strain and lower mutant ASFV infection, which could improve epidemiological surveillance, outbreak monitoring, and knowledge of the circulating ASFVs. In the future, attention should be paid to differentiate ΔCD2v infection from a vaccine immunization with HLJ/18-7GD. Of note, among the seven genes deleted from HLJ/18-7GD, we screened six deleted genes in the MGF domain for their antigenicity, none of which show high antigenicity in sera from survived pigs infected with wild-type ASFVs (data not shown), and only CD2v is immunogenic and appropriate for serological assay. Therefore, nucleic acid detection targeting the seven deleted genes, including the EP402R gene, and six genes in the MGF domain would likely be the best possible options and used together to distinguish lower virulent variant infection and HLJ/18-7GD vaccination. Although the newly established ELISAs were found to be highly comparable with the commercial ELISA kit, future studies are warranted to further validate the field use of these methods.

## Figures and Tables

**Table 1:** The sequence of primers used for PCR amplification of p54 and CD2v.

Target Gene	Primer	Sequence (5′–3′)	Amplicon Size (bp)
p54/E183L	pET-30a-p54F	AG GATATC ATG AGCAGCAGGAAGAAGAAGGCC	399
	pET-30a-p54R	CT CTCGAG CTACAGGCTGTTCTCCAGGTCCTTG	
CD2v/ EP402R	pAcHBM-CD2vF	GCGGATCCTACTGGGTGAGCTTCAACAAGACCATCATCCT	561
	pAcHBM-CD2vR	CGCGGCCGCTTCATGCCACTCAATCTTCTGAGCCTCGAAAATA	

Underlined nucleotides are the restriction sites included with the respective primer.

**Table 2:** Intra- and inter-assay repeatability test of the established ELISA.

Serum Samples	p54-ELISA				CD2v-ELISA			
	Intra-Assay		Inter-Assay		Intra-Assay		Inter-Assay	
	$\bar{X}$ ± SD	CV%	$\bar{X}$ ± SD	CV%	$\bar{X}$ ± SD	CV%	$\bar{X}$ ± SD	CV%
P1	0.81 ± 0.02	2.96	0.94 ± 0.11	11.8	0.963 ± 0.028	2.89	0.74 ± 0.07	10.08
P2	0.94 ± 0.05	5.12	1.01 ± 0.07	7.3	0.937 ± 0.034	3.64	0.69 ± 0.05	6.87
P3	0.83 ± 0.02	2.46	0.94 ± 0.11	12.0	0.768 ± 0.025	3.19	0.66 ± 0.09	13.61
P4	0.83 ± 0.06	7.27	0.92 ± 0.10	10.9	0.707 ± 0.029	4.04	0.53 ± 0.06	10.94
P5	0.95 ± 0.05	5.92	0.96 ± 0.06	6.5	0.987 ± 0.057	5.73	0.79 ± 0.09	11.16
N1	0.05 ± 0.06	10.31	0.05 ± 0.03	5.2	0.049 ± 0.003	6.005	0.057 ± 0.005	8.95
N2	0.05 ± 0.03	5.34	0.05 ± 0.01	1.9	0.054 ± 0.003	5.041	0.054 ± 0.006	11.98
N3	0.05 ± 0.05	8.66	0.055 ± 0.04	7.9	0.050 ± 0.004	7.633	0.052 ± 0.004	6.93
N4	0.19 ± 0.01	5.85	0.194 ± 0.01	5.7	0.098 ± 0.002	1.830	0.089 ± 0.005	5.67
N5	0.07 ± 0.06	8.44	0.079 ± 0.08	10.5	0.076 ± 0.003	3.758	0.069 ± 0.004	5.88

“P” indicates the known positive serum samples and “N” is for known negative samples.

## Data Availability

Not applicable.
